# Ninth Annual Meeting of the National Dental Association

**Published:** 1905-10

**Authors:** William H. Trueman


### NINTH ANNUAL MEETING OF THE NATIONAL DENTAL ASSOCIATION.

THE ninth annual meeting of the National Dental Association, held at Buffalo, N. Y., convened in the auditorium of the Central Y. M. C. A. building at ten o'clock, Tuesday morning, July 25, 1905, the President, Dr. Waldo E. Boardman, of Boston, Mass., in the chair.

Dr. Charles S. Butler, of Buffalo, delivered the address of welcome on behalf of the mayor of Buffalo, who was unable to be present.

The session was then opened by the President reading his address, which, after a short discussion, was referred to a committee.

The first business transacted was on a motion by Dr. H. J. Burkhardt, of Batavia, N. Y., to amend the constitution and by-laws, changing the basis of representation so as to admit to membership in the National Association delegates from State and Territorial societies only, thus restoring a basis which proved so unsatisfactory, after trial, early in the career of the organization, that it was promptly changed.

Notice of this was given last year, and it was brought up at this meeting as unfinished business. It provoked an angry discussion, took up much valuable time, and was finally passed by a small majority, and was made to take effect immediately, notwithstanding that the announcement for this session distinctly states that "Members of the profession are cordially invited to this meeting, and may join the association by presenting delegates' certificates from their State or local societies." In the face of this, making the amendment immediately effective was an outrageous proceeding, and placed a number who had come to the meeting with certificates from local societies in an embarrassing position. The writer suggests that in all future announcements the clause defining conditions of membership be immediately followed by the words, "*subject to change without notice,*" in conspicuous display type. This action is the more indefensible from the fact that it was not prompted by any desire to benefit the society, but for the purpose of keeping out a single individual who, having been expelled from the leading dental society in his locality, had gathered around him a sufficient number of associates to organize a new society, and came as their delegate. Rather than assume the responsibility of declaring this new society or its delegate unworthy, the majority of those present preferred that the association should place itself on record as inviting members of the profession to unite with it on terms distinctly stated, and then, after these had been complied with, and the candidates were present, without cause refusing them admission. The injustice of this was so evident that the Council advised that it be recalled. It is a dangerous thing for associations like this to take part in local quarrels, and unwise to change its general rules to meet special cases.

This was followed by a long report from the Committee on Army and Navy Legislation. Such long reports should always be printed and distributed, so that members may read them at their leisure. However interesting or important, or however well read, they are tiring to the audience, and very few indeed can follow their reading sufficiently well to fully understand their import.

This was followed by adjournment.

The afternoons and evenings were allotted, according to programme, to meetings of sections, and accommodations were prepared at Hotel Iroquois, head-quarters of the Association, for the three sections to meet in separate rooms, at the same time, for reading of papers and discussions. For an association so few in numbers as is the National, this is a very unsatisfactory arrangement. While the list of papers was long, very few in attendance heard many read. There was, in the first place, no certainty when any particular paper would be presented, so no one could select any one section without risk of disappointment; and the effort of some to overcome this caused a to-and-fro movement from one section to another that was distracting. Of the thirty-two papers on the programme, the greater number were, judging by their titles, not such as should be brought before a National Association. Some were, practically, second-hand; others, while good, were upon subjects far more suitable for local society discussion; others, again, were on subjects quite threadbare, and would find their proper place in an undergraduate student's society. Were these eliminated, and the time of the association properly utilized, all that remained could have been properly cared for in general sessions.

Very much time was wasted by not beginning the meetings of sections at the time appointed. By means of placards the various events were fairly satisfactorily announced, but (the evening meetings especially) they were not promptly called. Frequent changes were made, and the meetings of general sessions, of committees, and of sections, were very confusing. Not only was this time-wasting, but the delay and the uncertainty discouraged that keen interest in the proceedings so essential to a successful meeting. Members, after standing around half an hour or more waiting for a session to open, strolled off, to straggle in later, when the session was half over.

The preparation and proper presentation of scientific matter for information and discussion is admittedly a vexed question. The present scoop-net method is faulty. Selection by invitation, as the profession is now organized, would probably prove equally unsatisfactory. Any censorship, unless tactfully exercised, invites trouble. Should the Association succeed in launching a journal of its own, this might prove an acceptable outlet for papers prepared for the association meetings that in either subject or treatment were not deemed the best to bring before it. They need not be rejected; they could be read by title and accepted for the journal by a complimentary vote of thanks to the writers. Papers on subjects of general professional interests, education, legislation, etc., original research, matters pertaining to practice that are entirely new, seem to be much more appropriate for presentation to a National Association than variations of old methods or matters which have been frequently discussed.

Meetings conducted as was this, while a social success, fall far below what they might be in helpfulness to professional growth. There is nothing in them attractive to the new-comer, one who is not yet initiated into the inner circle. This is serious, for the new-comer is very important to a society's progress, indeed to its very existence. Without a constant influx of new life, of new ideas, and new ideals, the period of decadence is soon reached, and "race suicide" invited. Only those born later can keep in close touch with men and affairs of later days.

The multiplicity of fraternities, and of associations meeting at the same time and place as the National Association, and under its shadow and wing, is a growing evil. However worthy and useful in themselves, they are intruders and parasites when permitted to interfere with the more important body, as they undoubtedly did at Buffalo.

There was far too much disorder on the platform. During the opening session, at one time, all on the platform, except the member speaking, were engaged in an animated discussion among themselves. This is distracting alike to the speaker and to the audience. At the general sessions and the section meetings a constant running to the platform of this one and that kept up a commotion that was annoying to the speaker, and divided the attention of the audience. Such thoughtless actions detract very much from the dignity of the proceedings, and should be by all means avoided.

Enthusiasm is necessary to a thoroughly successful meeting of a body like this. That is best promoted by a series of properly conducted consecutive sessions, well attended. Vacant chairs are not inspiring.

No one individual could hear enough of either the papers or the discussions at Buffalo to pass judgment upon their merit, or to give an idea of their value, scope, or character. In that respect the meeting was unsatisfactory. The profession must wait for this perhaps a year or more, and accept it in driblets. It will come to them in time, like the "left-overs" of a forgotten feast—much of it insipid and useless. The same is true, in a manner, of the business transacted.

The manufacturers' exhibits lost much of their impressiveness by being displayed in a number of small rooms. This, however, to the writer seemed an advantage. It gave the attendants a much better opportunity to present their wares, and the dentist a much better opportunity to examine and select, than when jostled by a crowd. The exhibits were excellent, varied, and instructive. They were unobtrusive without being in the least degree suppressed. Of late, the tendency has been to permit commercial interests to occupy so prominent a place that professional interests have become a mere annex. It was not so at Buffalo.

The clinics, held the mornings of Wednesday and Thursday,

July 26 and 27, in the Dental Department building of the University of Buffalo, were the most satisfactory part of the meeting. The various rooms, and their furnishings afforded excellent accommodations. The clinics had been well advertised, and the supply of patients was abundant. Every thing was well ordered, and promptly and without confusion each day's work was commenced. Every opportunity was afforded onlookers at the operative clinics to inspect each critical stage of the operation as it was reached at the various chairs. This was an excellent feature. Having opportunity to examine the field of operation before anything was done, knowing what the operator proposed to do, again examining the cavity when ready to receive the filling, and having the special points of interest in its preparation pointed out, and later, under like favorable circumstances, being able to note the progressive steps by which the filling was constructed, and finally, at leisure, to critically inspect the finished work, made the operation interesting and instructive. This was done in all cases that admitted of it. It was more helpful, indeed, to the observer than to watch an operation from start to finish in that more than one operator's work was seen. It was helpful also to the operator; it relieved him of a distracting crowd when the work in hand most needed his undivided attention, and the intervals when his work was inspected gave him needed rest. As many like operations were going on at the same time, the onlookers were kept busy going from chair to chair in response to the announcement that so and so had reached an interesting stage of his work. To enter into a description of the operations performed is beyond the scope of this brief sketch. Suffice it to say that interest centred in the work of members of the G. V. Black Dental Club of St. Paul, exponents of the much talked of "extension for prevention" ideas, etc., of later days. There were other operators, but these attracted the most attention in the room in which the gold-workers were located.

The writer was impressed by the great change which has taken place in later days. A few years ago nearly all the gold operations here performed would have been done with an electric mallet. Not one was seen in the clinic. All the work was done with a hand mallet in the hands of a trained lady assistant. The business-like ways, the non-obtrusiveness, the precision of movement, the patient attentiveness of these lady assistants in anticipating and supplying the operators' needs was quite marked.

Nearly all the gold-workers were *sans* coat and vest, in "shirt sleeves," an attire out of place at a dental chair, even at a clinic. It is true that the room was warm; a light coat adds but little to one's discomfort, however, and looks far more professional than a day-laborer's garb. The "slouchy" positions some assumed while working were justly criticised; such positions are as unnecessary as they are unhealthy and ungraceful. Operators at a dental clinic should remember that they themselves are part of the exhibits, quite as much so as is their work, and conduct themselves accordingly.

Porcelain work held second place; it was not, however, neglected. A number of skilled operators, located in another room, by constructing and inserting practical work, demonstrated their methods and, at the same time, the distinctive features of several different electric furnaces used.

The new anæsthetics, narcotile and somnoforme, were used, practically, for tooth extraction; and in addition they were administered to any dentist who desired to personally experience their effects.

Several surgical clinics were on the programme, but as these were held at the Buffalo General Hospital, and at the same time as these clinics were going on, they were missed by the writer.

The table clinics were an interesting feature. Among those which especially attracted the writer's attention was a press exhibited by Dr. J. A. Brown, of Morrisville, Vt., especially designed for swaging aluminum plates. He claimed for this method not only ease of working and accuracy of adaptation, but a better plate, inasmuch as the hard surface given to sheet aluminum by rolling was not impaired. It may not be generally known that when aluminum is rolled into plate the surface immediately in contact with the rollers is condensed and thereby becomes more resistant to wear and to corrosion. Swaging between dies usually roughens this surface so that in the smoothing and polishing processes it is entirely removed. Dr. Brown claims that by the method he demonstrated this was avoided.

Dr. L. L. Bosworth, Toledo, Ohio, exhibited an electric furnace for gold soldering. This was not a muffle furnace. It was shaped somewhat like a round asbestos soldering-block; the depression, however, was deeper, and sufficiently large to hold a full denture invested and ready for soldering. This cavity, its bottom and sides fitted with thin platinum wire, as is the muffle of porcelain electric furnaces, becomes evenly heated when the current is turned on. In simple cases the blow-pipe may not be needed; in others it is used while the case is still in the furnace to direct the flow of the solder. It is claimed for this furnace, greater convenience, safety to the porcelain teeth, and less risk of the plate warping.

Dr. A. L. Haas, of Des Moines, Iowa, demonstrated an appliance for swaging gold or platinum caps to the end of tooth-roots prepared for crowning. All the work is done upon the root itself, in the mouth. The gold or platinum plate is roughly shaped to the root, and the dowel passed through. A punch, provided with a series of removable faces to suit different sizes of tooth-roots, with a hole loosely fitting the dowel, is then adjusted over it, with a piece of unvulcanized rubber between the plate and the face of the punch. A sharp blow with a mallet on the end of the punch drives the plate into close contact with the root, and marks its outline. It is now removed, and the dowel soldered to the plate. The edges of the plate are now bent so as to fit over the root, and so form a seamless cap fitting over the face of the root and extending rootward as far as desired. This is fitted to the root and the punch applied as before, to correct any change made by soldering and bending. It seemed to be a practical idea.

Dr. W. B. Dunning, of New York City, demonstrated a method of packing non-cohesive gold that to the writer was quite new. He formed the gold into small, soft, almost square pellets, and packed them into position, using an instrument with a sharp edge, very like a large hatchet excavator. This edge was kept quite sharp and smooth by frequent application to an Arkansas stone. The doctor especially recommended the method for small, irregular cavities. The method was not rapid; it made, however, a solid, very hard filling, that finished as nicely as though of molten gold.

Dr. L. C. Taylor, of Hartford, Conn., showed how to combine gold and cement. The cavity need not be prepared so carefully for retention as is usual when gold alone is used. The cement, he mixed to about the same consistency as it would be to retain an inlay, and with it partially filled the cavity. He then took a pellet of crystal gold large enough to nearly fill the cavity, heated this quite hot, and immediately pressed it into the cement. By heating the gold the air in its interstices is expanded, as this contracts when it is forced into the cement, the cement is drawn up into it, making a more reliable union between the two than would otherwise take place. The heat also hastens the hardening of the cement. The gold is held firmly in position until the cement hardens. It is then packed, more added if needed, and finally well burnished and finished. Dr. Taylor has made free use of this method in filling children's teeth, fissure cavities where he did not desire to extensively excavate, and in cases where an easily introduced filling is called for. He found fillings so inserted satisfactory and durable. He laid particular stress upon having the gold quite hot when it is pressed into the cement.

An interesting exhibit, occupying a small room, was made by the Dental Department of Harvard. In the collection the writer observed a series of lantern slides, such as are used in class instruction; instruments used by the students in making dental alloys, and for testing the expansion and contraction of amalgam; and several microscopes with slides showing specimens of crystals brought to professional notice by recent researches regarding saliva. Crystals from saliva and crystals of corresponding salts artificially produced were shown side by side. The collection was well selected, to show methods and means of instruction employed in the Dental Department. It would be a good idea if the colleges generally embraced the opportunity afforded by these large professional gatherings to show advanced methods for imparting instruction now in use. So few dentists ever enter a college after they graduate that it is not generally known how much change has been made of late years in their methods and means.

Eighty-five clinics were on the programme for each day. One hesitates to criticise, especially after seeing an exhibit, that seemed too trifling for notice, surrounded hour after hour by an interested crowd; yet, looking over the list, an impression remains that a judicious pruning would have done no harm; not that there was anything objectionable, but rather a lack of novelty and importance in many of the table clinics, and in some of the chair clinics. It seems a useless waste of time to demonstrate matters that have been accepted facts for many years.

The number in attendance was said to be very satisfactory; it was less, however, than the writer recalls at meetings of the American Association of more than a score of years ago. The conditions of membership have not been changed, while the number of practising dentists, the contributing societies, and the membership therein have very materially increased. This is not encouraging. This would have no meaning if the National Association was a strictly representative body. This is not the case. It is very largely composed of permanent members who owe no allegiance to the body through which they came to the Association. To a local society it is a matter of no moment whether all of its members or none are connected with the National Association, and makes no difference whether its appointed delegates attend or not. From one year's end to another it is very seldom, indeed, that anything occurs to remind the members of a local society that there is in the United States a National Dental Association, and equally rare that the local societies are referred to, helpfully, in the National Association proceedings. The National Association does not concern itself in making known its doings to the profession at large, and is practically unknown. At Buffalo, the section best attended was one not on the programme, the social section, almost continuously in session in the hotel lobby. To its own members accustomed to meet year after year, and to those well known to its members, it was very interesting, enjoyable, and will be longest remembered. To the "young fry," however, while it may have proved interesting and instructive,—far more so than attractive,—it is not likely that it tended to an increase of membership. They were left out in the cold. There seems to be a need for reorganization on lines better suited to geographical conditions, a reorganization which shall definitely define the position, the work, and the relations to each other of local, State, and national bodies; a reorganization from which the permanent membership idea is entirely eliminated in all but the local societies. They can then work together, and form an harmonious, compact body commanding the respect and support of the profession at large.

William H. Trueman.

